# In-Situ Measurement of Electrical-Heating-Induced Magnetic Field for an Atomic Magnetometer

**DOI:** 10.3390/s20071826

**Published:** 2020-03-25

**Authors:** Jixi Lu, Jing Wang, Ke Yang, Junpeng Zhao, Wei Quan, Bangcheng Han, Ming Ding

**Affiliations:** 1 Research Institute for Frontier Science, Beihang University, Beijing 100191, China; lujixi@buaa.edu.cn (J.L.); quanwei@buaa.edu.cn (W.Q.); 2 Hangzhou Innovation Institute, Beihang University, Hangzhou 310051, China; hanbangcheng@buaa.edu.cn; 3 School of Instrumentation and Optoelectronic Engineering, Beihang University, Beijing 100191, China; wangjing_vicky@buaa.edu.cn (J.W.); yangke@buaa.edu.cn (K.Y.); junpengvzhao@gmail.com (J.Z.)

**Keywords:** electrical heating, in-situ measurement, atomic magnetometer, spin-exchange relaxation-free

## Abstract

Electrical heating elements, which are widely used to heat the vapor cell of ultrasensitive atomic magnetometers, inevitably produce a magnetic field interference. In this paper, we propose a novel measurement method of the amplitude of electrical-heating-induced magnetic field for an atomic magnetometer. In contrast to conventional methods, this method can be implemented in the atomic magnetometer itself without the need for extra magnetometers. It can distinguish between different sources of magnetic fields sensed by the atomic magnetometer, and measure the three-axis components of the magnetic field generated by the electrical heater and the temperature sensor. The experimental results demonstrate that the measurement uncertainty of the heater’s magnetic field is less than 0.2 nT along the *x*-axis, 1.0 nT along the *y*-axis, and 0.4 nT along the *z*-axis. The measurement uncertainty of the temperature sensor’s magnetic field is less than 0.02 nT along all three axes. This method has the advantage of measuring the in-situ magnetic field, so it is especially suitable for miniaturized and chip-scale atomic magnetometers, where the cell is extremely small and in close proximity to the heater and the temperature sensor.

## 1. Introduction

Atomic magnetometers have been used in a wide range of applications to detect weak magnetic fields, such as magnetoencephalography (MEG) [1,2,3], magnetocardiography (MCG) [4,5,6], and the search for new physics [7,8]. Currently, the most sensitive atomic magnetometer is the spin-exchange relaxation-free (SERF) magnetometer [9], which was first presented by the Romalis group at Princeton University [10]. It has outperformed SQUIDs (superconducting quantum interference devices) and has the advantage of noncryogenic operation. However, the SERF magnetometer needs to operate in a very small magnetic field, and its vapor cell should be heated to very high temperatures (usually 100–200 ${}^{{}^{\circ}}$C, depending on the kind of alkali metal) [11,12,13,14]. The heating elements must supply enough power to maintain the cell temperature without introducing magnetic noise that could affect the magnetometer’s performance.

There are essentially three kinds of heating techniques used for SERF atomic magnetometers, as well as other sensitive atomic sensors: hot air heating [15,16,17,18], optical heating [4,19,20,21] and electrical heating [9,22,23,24,25]. Among these heating techniques, electrical heating is most flexible and efficient, and thus widely used in all kinds of atomic sensors. However, the heating current inevitably produces a magnetic field, which has detrimental effects on ultrasensitive magnetometers. In order to suppress this magnetic field interference, two methods have been widely used that have proved to be effective. The first method is to modulate the driving currents at high-frequency, which can shift the heating-induced magnetic field beyond the magnetometer’s bandwidth. If the amplitude of the magnetic field is too large, it will still affect the magnetometer on spin-exchange relaxation and sensitivity [26]. The second method is to configure the heating wire twisted or back-to-back on a two-layer film, to cancel the magnetic field induced by the heating current. Because the current through adjacent wires cannot overlap completely, the amplitude of the magnetic field cannot be suppressed sufficiently, and low-frequency magnetic noise can still interfere with the measurement. Therefore, these two methods are usually used in combination with each other [9,22,23,24,25]. It can be seen that accurate measurement of the amplitude of electrical-heating-induced magnetic field is important, which could help estimate the electrical heater’s effect and guide the improvement of it.

Although many studies on atomic magnetometers concern the electrical heating technique, most of them evaluate its performance by directly observing the signal or the sensitivity of the magnetometer [27,28,29,30,31]. This is not a quantitative method, and thus there is little guidance for the design and improvement of the heating elements. Only a few researchers have measured the heating-induced magnetic field using commercial magnetometers. Yim et al. designed a double-layer film heater and measured the magnetic field of it using a commercial fluxgate [32]. Liang et al. developed a small heating chip, and measured its induced magnetic field by a commercial atomic magnetometer [33]. This kind of method works well unless the measured uniform region is significantly larger than the probe of the magnetometer. However, for the miniaturized and chip-scale atomic magnetometer, the heater is very close to the cell and the heating-induced magnetic field is not uniform. Therefore, using this kind of method, the actual magnetic field in the location of the sensitive atoms cannot be accurately measured. Moreover, existing methods have not yet taken into account the magnetic field generated by the temperature sensor of the heater, which is also a potential source of magnetic field interference for the magnetometer.

In this paper, we propose a novel method for in-situ measurement of the amplitude of the three-axis magnetic field generated by the heater and the temperature sensor for a SERF atomic magnetometer based on the magnetic field zeroing technique. This method only requires its own vapor cell for the measurement, and does not need additional magnetometers. As a result, this method is not limited by the volume of the atomic magnetometer. First, the three-axis magnetic field sensed by the SERF atomic magnetometer was compensated in the DC and AC heating modes, respectively. The compensation values in these two modes were subtracted to obtain the amplitude of the magnetic field generated by the heater. Next, we conducted this process at different cell temperatures, and accordingly obtained the amplitude of the magnetic field with different driving currents. At last, we compensated the three-axis magnetic field in the AC heating mode for different currents passing through the temperature sensor, and extracted the magnetic field generated by the temperature sensor.

## 2. Method

**Figure 1:**
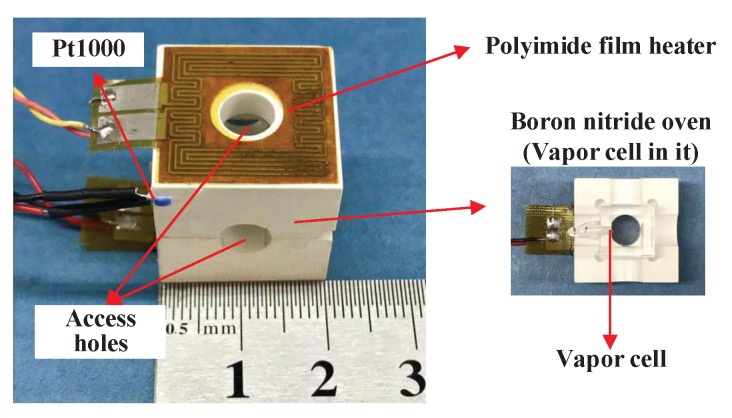
Photograph of the vapor cell and the heating components of an spin-exchange relaxation-free (SERF) atomic magnetometer.

Figure 1 shows the vapor cell and the heating components of a SERF atomic magnetometer. A square vapor cell with 10 mm in length, containing a droplet of potassium metal, 650 torr ${}^{4}$He, and 50 torr N${}_{2}$, was fixed inside a two-part boron nitride oven. There were four access holes in the oven for the pump and probe beams. Two pieces of double-layer polyimide film heaters, glued to the oven’s upper and lower surfaces respectively, were wired in series and driven by a homemade power amplifier circuit. The resistance wires in the polyimide film heaters were made of non-magnetic constantan. Each heater had a resistance of 57.2 Ω and was a 19 × 19 mm${}^{2}$ square, with an access hole and a protrusion of the heater leads. Two layers of each heater were patterned back-to-back and wired in series, and the current directions of adjacent wires on each layer were opposite to one another. The width and thickness of the wire was 0.32 mm and 0.01 mm, respectively. The separation of the layers was 0.12 mm. The magnetic field generated by the current through the wire on the lower layer was canceled significantly by that on the upper layer. A commercial Pt1000 temperature sensor (L420, class A, Heraeus, Hanau, Germany) with non-magnetic AgPd wires was glued to the oven. Its resistance was measured using the four-wire configuration, and a constant current was applied to the temperature sensor. As the magnetometer operated, the cell was heated to a set temperature by closed-loop driving of the heater. The heater and temperature sensor-generated magnetic fields owing to the driving currents through them, which were sensed by the atomic spins in the cell.

The behavior of the atomic spin vector ***S*** of the SERF atomic magnetometer can be described by a Bloch equation [16,34]:

(1) $$\frac{d}{dt}S=\frac{1}{q}\left[\gamma_{e}B\times S+R_{\mathrm{op}}\left(\frac{1}{2}\hat{z}-S\right)-R_{\mathrm{rel}}S\right],$$

where *q* is the slowing-down factor, *γ_e_* is the electron gyromagnetic ratio, ***B*** is the magnetic field vector (*B_x_*, *B_y_*, *B_z_*) sensed by the atomic spins, *R*_op_ is the pumping rate along the *z* axis, and *R*_rel_ is the spin-relaxation rate.

During the operation, the SERF atomic magnetometer would be enclosed in magnetic shields. Based on Equation (1), in small magnetic fields, the steady-state response of the atomic spins along the *x* axis to a small magnetic field can be described as

(2) $$S_{x}=\frac{R_{\mathrm{op}}}{2R_{\mathrm{tot}}}\left[\frac{\gamma_{e}}{R_{\mathrm{tot}}}B_{y}+{\left(\frac{\gamma_{e}}{R_{\mathrm{tot}}}\right)}^{2}B_{x}B_{z}\right],$$

where $R_{\mathrm{tot}}$=$R_{\mathrm{op}}$+$R_{\mathrm{rel}}$.

Based on Equation (2), the three components of the quasi-static magnetic field can be orderly compensated to zero using the magnetic field zeroing technique described in [27,35] as:

(1) Apply a small and low-frequency oscillating field $B_{o}\cos\left(\omega t\right)$ along the *z* direction and compensate $B_{x}$, until the response of $S_{x}$ to $B_{o}\cos\left(\omega t\right)$ is a minimum.

(2) Apply a small and low-frequency oscillating field $B_{o}\cos\left(\omega t\right)$ along the *x* direction and compensate $B_{z}$ until the response of $S_{x}$ to $B_{o}\cos\left(\omega t\right)$ is a minimum.

(3) Compensate $B_{y}$ until the DC output of $S_{x}$ is zero.

(4) Repeat the process (1) to (3) until the compensating magnetic fields in all three axes are stable.

The oscillating fields and the compensation magnetic fields in the process above are produced by a set of three-axis coils in the experiment. After the compensation, the magnetometer is most sensitive to the magnetic field along the *y* axis. The magnetic field B sensed by the atomic spins contains four components: the residual magnetic field in the magnetic shields $B^{r}$, the coils’ magnetic field $B^{c}$, the heater’s magnetic field $B^{h}$, and the temperature sensor’s magnetic field $B^{t}$, which can be expressed as

(3) $$B=B^{r}+B^{c}+B^{h}+B^{t}.$$

In high-performance magnetic shields, the residual magnetic field $B^{r}$ is stable. $B^{c}$ is zero before the compensation. $B^{h}$ and $B^{t}$ are proportional to the current through the heater and the temperature sensor, respectively. The current through the temperature sensor is constant, and thus $B^{t}$ will be fixed. The driving current of the heater is determined by the heating power, which is related to the cell temperature, the environmental temperature and the thermal insulation.

If the heater is driven by a DC current (DC heating mode), using the magnetic field zeroing technique, B would be compensated to zero by the coils and we can get

(4) $$B_{}^{c\_\mathrm{DC}}=-\left(B^{r}+B^{t}+B_{}^{h\_\mathrm{DC}}\right),$$

where $B_{}^{c\_\mathrm{DC}}$ and $B_{}^{h\_\mathrm{DC}}$ are the coils’ compensation magnetic field and the heater’s magnetic field in the DC heating mode, respectively.

When the driving current is modulated at a frequency much higher than the magnetometer’s bandwidth, the magnetometer cannot respond to this high-frequency AC magnetic field ($B_{}^{h\_\mathrm{AC}}$). Using the magnetic field zeroing technique, B would be compensated to zero by the coils and thus we can get

(5) $$B_{}^{c\_\mathrm{AC}}=-\left(B^{r}+B^{t}\right),$$

where $B_{}^{c\_\mathrm{AC}}$ is the coils’ compensation magnetic field in the AC heating mode.

By subtracting Equation (5) from Equation (4), $B_{}^{h\_\mathrm{DC}}$ can be obtained by

(6) $$B_{}^{h\_\mathrm{DC}}=B_{}^{c\_\mathrm{AC}}-B_{}^{c\_\mathrm{DC}}.$$

It should be noted that under the same heating condition, the magnitude of $B_{}^{h\_\mathrm{DC}}$ is the same with the RMS value of $B_{}^{h\_\mathrm{AC}}$. Therefore, the magnitude of $B_{}^{h\_\mathrm{AC}}$ can be obtained according to $B_{}^{h\_\mathrm{DC}}$ and the modulation waveform. Moreover, we can measure the scale factor between the driving current $I_{h}$ and $B_{}^{h\_\mathrm{DC}}$. Accordingly, for the same magnetometer and heating conditions, the magnetic field generated by the heater at other cell temperatures can be easily obtained by measuring the driving current.

Afterwards, in the AC heating mode, we can modify the excitation current of the temperature sensor $I_{t}$, and $B^{t}$ will change linearly as $B^{t}=k^{t}I_{t}$. Here, $k^{t}=\left(k_{x}^{t},k_{y}^{t},k_{z}^{t}\right)$ is the scale factor between $I_{t}$ and $B^{t}=\left(B_{x}^{t},B_{y}^{t},B_{z}^{t}\right)$. Therefore, Equation (5) can be expressed using

(7) $$B_{}^{c\_\mathrm{AC}}=-\left(B^{r}+k^{t}I_{t}\right).$$

At different excitation currents $I_{t}$, we compensate the three-axis magnetic field, and accordingly calculate $k^{t}$ and $B^{r}$ using a linear fitting based on Equation (7). Further, $B^{t}$ can be obtained by $k^{t}I_{t}$.

## 3. Experimental Setup and Procedure

**Figure 2:**
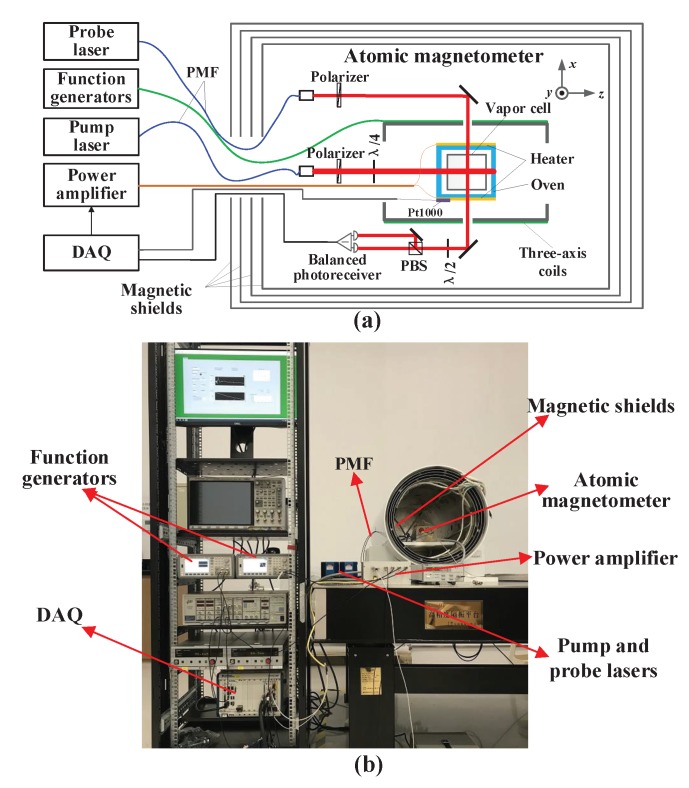
Schematic (**a**) and photograph (**b**) of the experimental setup of the SERF atomic magnetometer. PMF: polarization maintaining optical fiber; DAQ: data acquisition.

The experimental setup was a typical SERF atomic magnetometer with an orthogonal pump-probe arrangement (Figure 2). Potassium atoms in the vapor cell were pumped by a circularly polarized beam propagating along the *z* axis tuned on the center of the D1 line (770.108 nm). The *x* component of potassium atomic spin ($S_{x}$) caused by the magnetic field was measured using a linearly polarized laser detuned 60 GHz from the center of the D2 line (766.701 nm). The optical rotation angle proportional to $S_{x}$ was measured using a balanced polarimeter. Both pump and probe lights were originated by external cavity diode laser systems with optical fiber-coupled output, and sent to the magnetometer through the polarization-maintaining optical fiber (PMF). The diameters of the pump and probe beam were 4 mm and 2 mm, respectively. Therefore, the sensitive volume of the magnetometer was about ϕ2 mm × 4 mm. Four nested cylindrical μ-metal magnetic shields were utilized to attenuate the external magnetic field. A set of three-axis coils were driven by function generators. Radial (the *x* axis and the *y* axis) and longitudinal (the *z* axis) magnetic fields were generated by saddle coils and Lee-Whiting coils, respectively [36,37]. Some resistances were connected in series between the function generators and the coils, and the scale factor between the output voltage of function generators and the generated magnetic fields were adjusted to be 10 pT/mV along all three axes. The output resolution of the function generator was 0.1 mV, and thus the resolution of the applied magnetic field was 1 pT. A set of data acquisition (DAQ) was used to acquire the response of the magnetometer and control the cell temperature.

**Figure 3:**
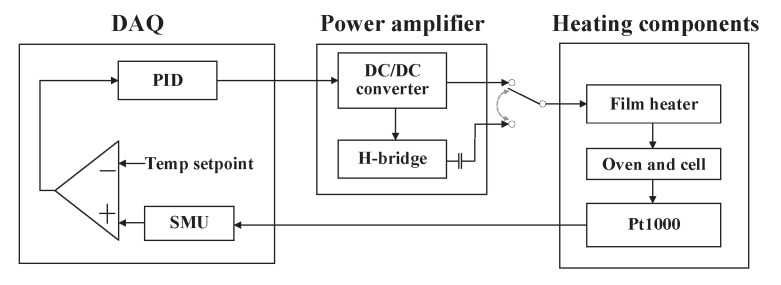
Schematic of the heating system of the atomic magnetometer. Temp: temperature; SMU: source measure unit.

Figure 3 shows the schematic of the heating system of the experimental setup. The vapor cell and the heating components in it are described in Section II and shown in Figure 1. The film heaters were parallel to the yz–plane. The resistance of the Pt1000 on the oven was measured using the four-wire configuration by a source measure unit (PXIe-4145, National Instruments, USA). A PID temperature controller, conducted by a LabVIEW VI, controlled a homemade power amplifier to drive the heater. The VI was written based on the “PID and Fuzzy Logic Toolkit” of National Instruments. The proportional and integral parameters were determined by the autotuning algorithm integrated in the toolkit, whereas the derivative action was not used. The power amplifier contained a DC/DC converter and an H-bridge circuit. The DC/DC converter circuit was constructed based on the chip TPS54160 (Texas Instruments, USA), and the H-bridge circuit was constructed mainly by the chip ISL83204A (Intersil, USA) and four MOSFETs. The former provided an output voltage regulated by the PID temperature controller, and the latter was used to modulate the output to be an AC square voltage at 43.2 kHz. The heater could be switched between DC and AC output of the power amplifier. The H-bridge circuit only switched the polarity of a voltage applied to the heater, and thus the amplitude of the DC voltage and the AC square voltage were equal. A capacitor was used to block the DC offset and the low-frequency noise further.

First, we heated the cell to 150 ${}^{{}^{\circ}}$C in the AC heating mode, and measured $B_{}^{c\_\mathrm{AC}}$ using the magnetic field zeroing technique. Next, the heating mode was switched to the DC heating mode. After several minutes when the cell temperature was stable, $B_{}^{c\_\mathrm{DC}}$ was measured using the same method, and $B_{}^{h\_\mathrm{DC}}$ was calculated using Equation (6). During this process, the excitation current of the Pt1000 was set at a constant value of 100 μA. The applied oscillating magnetic field along the *x* axis and the *z* axis was 1 nT@2Hz. Afterwards, we repeated this process at several other temperatures (160 ${}^{{}^{\circ}}$C, 170 ${}^{{}^{\circ}}$C, 180 ${}^{{}^{\circ}}$C, 190 ${}^{{}^{\circ}}$C), and measured the corresponding driving voltage by a multimeter. The driving current was calculated using the driving voltage and the resistance of the film heater. At last, the cell was set to be heated to 170 ${}^{{}^{\circ}}$C in the AC heating mode. We modified the excitation current of the Pt1000 (50 μA–450 μA) and measured corresponding $B_{}^{c\_\mathrm{AC}}$. Then $k^{t}$ was calculated using a linear fitting. Accordingly, we were able to using Equation (7) to obtain the temperature sensor’s magnetic field $B^{t}$ at different excitation currents.

## 4. Experimental Results and Discussion

Table 1 shows the coils’ compensation magnetic field in the AC heating mode ($B_{}^{c\_\mathrm{AC}}$) and in the DC heating mode ($B_{}^{c\_\mathrm{DC}}$) at different cell temperatures. The table shows that the variation of mean value of $B_{}^{c\_\mathrm{AC}}$ at different cell temperatures was very small (less than 0.01 nT along the *x* axis, 0.06 nT along the *y* axis, and 0.14 nT along the *z* axis), whereas the amplitude of $B_{}^{c\_\mathrm{DC}}$ increased steadily with cell temperature. This indicates that $B^{r}$ and $B^{t}$ were relatively stable during the period of the experiment, and the amplitude of $B_{}^{h\_\mathrm{DC}}$ increased with the driving current. When the ambient temperature fluctuated, the driving current of the heater would variate accordingly to keep the cell temperature stable. This variation induced a significant low-frequency fluctuation of the heater’s magnetic field in the DC heating mode. Therefore, the uncertainty of the compensation magnetic field in the DC heating mode was significantly larger than that in the AC heating mode, especially along the *y* axis. Table 2 shows the driving voltage, driving current, and the amplitude of three-axis magnetic fields generated by the heater at different cell temperatures. The amplitude of the three-axis components of $B_{}^{h\_\mathrm{DC}}$ ($\left|B_{x}^{h\_\mathrm{DC}}\right|$, $\left|B_{y}^{h\_\mathrm{DC}}\right|$, and $\left|B_{z}^{h\_\mathrm{DC}}\right|$) were calculated from the data in Table 1 using Equation (6). We can see that the measurement uncertainty of $B_{}^{h\_\mathrm{DC}}$ was less than 0.2 nT along the *x* axis, 1.0 nT along the *y* axis, and 0.4 nT along the *z* axis at all cell temperatures.

**Figure 4:**
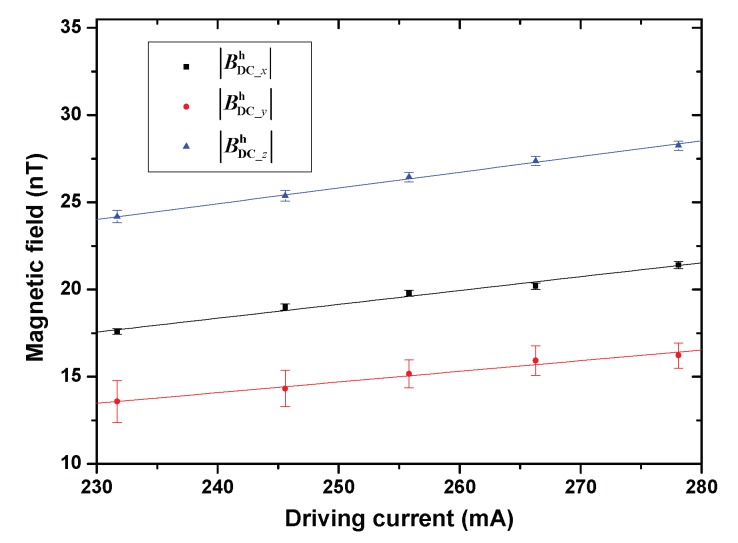
The amplitude of three-axis components of $B_{}^{h\_\mathrm{DC}}$ ($\left|B_{x}^{h\_\mathrm{DC}}\right|$, $\left|B_{y}^{h\_\mathrm{DC}}\right|$, and $\left|B_{z}^{h\_\mathrm{DC}}\right|$) as a function of the heater’s driving current ($I_{h}$). The lines that overlay the data is a linear fitting to $y=aI_{h}+b$. The fitting results are shown in Table 3.

Figure 4 shows $\left|B_{x}^{h\_\mathrm{DC}}\right|$, $\left|B_{y}^{h\_\mathrm{DC}}\right|$, and $\left|B_{z}^{h\_\mathrm{DC}}\right|$ as a function of the driving current. The lines that overlay the data are a linear fitting to $aI_{h}+b$, which shows a good linear relationship. *a* represents the generated magnetic field of the heater per unit current. Table 3 shows *a* and *b* derived from the linear fitting. Based on the fitting results, for the same heater and heating condition, the magnetic field generated by the heater can be calculated by measuring the driving current or voltage. The nonzero intercepts of the fitting results indicate a nonzero magnetic field error when the heating current is extrapolated to zero. We think this error is mainly due to the fact that the driving current range in the measurements was much greater than zero, which induces a significant extrapolation error.

**Figure 5:**
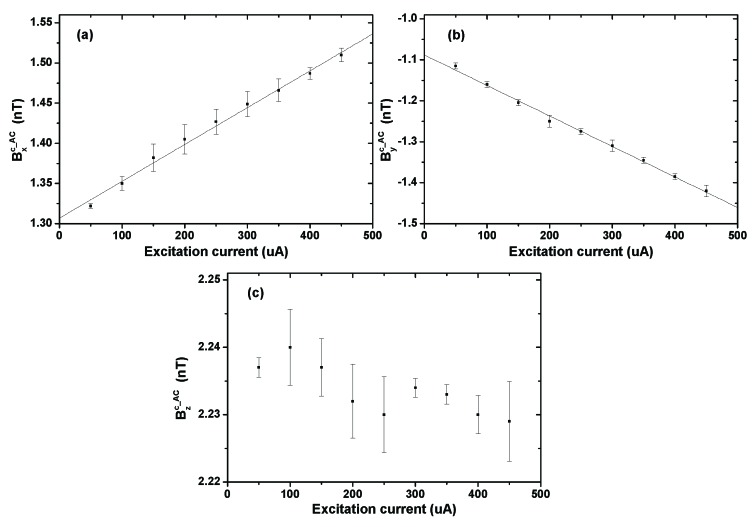
Three-axis components of $B_{}^{c\_\mathrm{AC}}$ as a function of the excitation current of the Pt1000 sensor. $B_{}^{c\_\mathrm{AC}}=(B_{x}^{c\_\mathrm{AC}}$, $B_{y}^{c\_\mathrm{AC}}$, $B_{z}^{c\_\mathrm{AC}})$. (**a**) $B_{x}^{c\_\mathrm{AC}}$, (**b**) $B_{y}^{c\_\mathrm{AC}}$, (**c**) $B_{z}^{c\_\mathrm{AC}}$. The lines that overlay the data is a linear fitting. The intercepts of the fitting results in (**a**) and (**b**) is 1.31 nT and −1.09 nT, respectively. The uncertainty of each measurement value is less than 0.02 nT.

When the cell was heated to 170 ${}^{{}^{\circ}}$C in the AC heating mode, the three-axis components of $B_{}^{c\_\mathrm{AC}}$ were measured as a function of the excitation current $I_{t}$ of the Pt1000. Based on Equation (7), $B_{}^{c\_\mathrm{AC}}$ should be linear with $I_{t}$. The measurement results are shown in Figure 5. It shows that $B_{x}^{c\_\mathrm{AC}}$ and $B_{y}^{c\_\mathrm{AC}}$ have a good linear relationship with the excitation current, whereas $B_{z}^{c\_\mathrm{AC}}$ does not show any obvious relationship. This may be because the Pt1000’s magnetic field had a very small component along the *z* axis of the magnetometer due to its relative position and orientation. The measurement uncertainty of the temperature sensor’s magnetic field is less than 0.02 nT along all three axes. The fitting results of the scale factors $\left|k_{x}^{t}\right|$ and $\left|k_{y}^{t}\right|$ are 0.458 nT/mA and 0.745 nT/mA, respectively. They represent the generated magnetic field of the temperature sensor per unit current. These two factors are nearly one order of magnitude larger than that of the film heaters (*a*), because the wires of the Pt1000 were not designed to a configuration that enabled the self-offset of the magnetic field. The intercepts of the fitting results indicate the residual magnetic field in the magnetic shield (1.31 nT along the *x* axis and −1.09 nT along the *y* axis). Therefore, when the excitation current was 100 μA, $B_{x}^{t}$ and $B_{y}^{t}$ should be 0.05 nT and −0.07 nT, respectively, and $B_{z}^{t}$ was estimated to be close to zero.

The experimental results above can help quantitatively estimate the magnetic noise of the heater and the temperature sensor, and guide their improvement. For example, according to the obtained linear relationship between the driving current (or voltage) and the heater’s magnetic field, the driving voltage noise can be measured, and then the magnetic noise generated by the heater can be calculated. If this noise exceeds other noises of the magnetometer, the configuration of the heater wire or the power amplifier noise should be further optimized. Using the same thought above, the magnetic noise generated by the temperature sensor can also be calculated. If the temperature sensor’s noise exceeds the requirement, we can modulate the excitation current at a frequency above the magnetometer’s bandwidth, or increase the distance between it and the cell.

## 5. Conclusions

In this paper, we propose an in-situ measurement method of three-axis magnetic fields generated by the electrical heater and the temperature sensor. Compared with existing methods, this method can be implemented by the atomic magnetometer itself, and thus does not need any extra magnetometers. The experimental results demonstrate that the measurement uncertainty of the heater’s magnetic field is less than 0.2 nT along the *x* axis, 1.0 nT along the *y* axis, and 0.4 nT along the *z* axis. The measurement uncertainty of the temperature sensor’s magnetic field is less than 0.02 nT along all three axes. This method can help quantitatively estimate the effects of the heater and the temperature sensor on the atomic magnetometer, and especially suitable for the miniaturized and chip-scale atomic magnetometers. Moreover, this method can also be used to measure the distribution of heating-induced magnetic fields by modifying the position of the probe light.

## Figures and Tables

**Table 1:** Compensation magnetic field in the AC heating mode ($B_{\mathrm{AC}}^{c}$) and in the DC heating mode ($B_{\mathrm{DC}}^{c}$) at different cell temperatures. The sign indicates the direction of magnetic field. $B_{\mathrm{AC}}^{c}=\left(B_{x}^{c\_\mathrm{AC}},B_{y}^{c\_\mathrm{AC}},B_{z}^{c\_\mathrm{AC}}\right)$; $B_{\mathrm{DC}}^{c}=\left(B_{x}^{c\_\mathrm{DC}},B_{y}^{c\_\mathrm{DC}},B_{z}^{c\_\mathrm{DC}}\right)$.

Temperature (${}^{{}^{\circ}}$C)	$B_{x}^{c\_\mathrm{AC}}$(nT)	$B_{y}^{c\_\mathrm{AC}}$(nT)	$B_{z}^{c\_\mathrm{AC}}$(nT)	$B_{x}^{c\_\mathrm{DC}}$(nT)	$B_{y}^{c\_\mathrm{DC}}$(nT)	$B_{z}^{c\_\mathrm{DC}}$(nT)
150	1.35 ± 0.01	−1.18 ± 0.20	−2.19 ± 0.01	18.94 ± 0.15	12.4 ± 0.8	22.00 ± 0.35
160	1.35 ± 0.01	−1.12 ± 0.22	−2.23 ± 0.01	20.34 ± 0.17	13.2 ± 0.8	23.15 ± 0.30
170	1.35 ± 0.01	−1.16 ± 0.22	−2.14 ± 0.01	21.14 ± 0.16	14.0 ± 0.6	24.21 ± 0.27
180	1.35 ± 0.01	−1.12 ± 0.20	−2.27 ± 0.01	21.54 ± 0.18	14.8 ± 0.6	25.10 ± 0.25
190	1.34 ± 0.01	−1.12 ± 0.20	−2.28 ± 0.01	22.74 ± 0.19	15.1 ± 0.5	25.98 ± 0.25

**Table 2:** The driving voltage, driving current, and the amplitude of three-axis magnetic fields generated by the heater. $B_{}^{h\_\mathrm{DC}}=\left(B_{x}^{h\_\mathrm{DC}},B_{y}^{h\_\mathrm{DC}},B_{z}^{h\_\mathrm{DC}}\right)$.

Temperature (${}^{{}^{\circ}}$C)	Driving Voltage (V)	Driving Current (mA)	$\left|B_{x}^{h\_\mathrm{DC}}\right|$ (nT)	$\left|B_{y}^{h\_\mathrm{DC}}\right|$ (nT)	$\left|B_{z}^{h\_\mathrm{DC}}\right|$ (nT)
150	26.53	232	17.59 ± 0.16	13.6 ± 1.0	24.19 ± 0.36
160	28.12	246	18.99 ± 0.18	14.3 ± 1.0	25.38 ± 0.31
170	29.29	256	19.79 ± 0.17	15.2 ± 0.8	26.45 ± 0.28
180	20.49	267	20.19 ± 0.19	15.8 ± 0.8	27.37 ± 0.26
190	31.84	278	21.41 ± 0.20	16.2 ± 0.7	28.26 ± 0.26

**Table 3:** The driving voltage, driving current, and the amplitude of three-axis magnetic field generated by the heater.

Case	*a* (nT/mA)	*b* (nT)
$\left|B_{x}^{h\_\mathrm{DC}}\right|$	0.079	−0.51
$\left|B_{y}^{h\_\mathrm{DC}}\right|$	0.060	−0.21
$\left|B_{z}^{h\_\mathrm{DC}}\right|$	0.090	3.25
